# Impact of Ethanol on Electrostatic Behaviour of Fluorocarbon Pharmaceutical Propellants

**DOI:** 10.3390/ph18111755

**Published:** 2025-11-18

**Authors:** Lochana Ranatunge, Manoochehr Rasekh, Hussein Ahmad, Wamadeva Balachandran

**Affiliations:** College of Engineering, Design and Physical Sciences, Brunel University of London, Uxbridge UB8 3PH, UK

**Keywords:** propellants, triboelectric charging, dielectric, electrostatic, polymer

## Abstract

**Background/Objectives:** Triboelectrification in fluid systems, and specifically in hydrofluorocarbon (HFC)-based propellants, used in pressurised metered-dose inhalers (pMDIs) remains understudied despite its impact on aerosol behaviour and does delivery. This study investigates how ethanol concentration affects charge generation and dissipation in HFC-152a (1,1-difluoroethane; R152a) flowing through low-density polyethylene (LDPE) tubing, a common valve-stem material in pMDIs. **Methods:** Controlled experiments measured electrical current, charge accumulation, and flow stability for HFC-152a with varying ethanol concentrations in LDPE tubing. Statistical analysis (two-way ANOVA, *p* < 0.05) assessed the effects of the propellant and material. Comparative tests include R134a (1,1,1,2-tetrafluoroethane) and R227ea (1,1,1,2,3,3,3-heptafluoropropane), and the tubing materials are polybutylene terephthalate (PBT), polyvinyl chloride (VINYL), polyoxymethylene (POM), and LDPE. **Results:** Increasing ethanol concentration produced larger measured currents, reduced net charge accumulation, and improved flow stability; these effects are attributed to ethanol’s higher dielectric constant and conductivity enhancing charge mobility and dissipation. Significant propellant x material interactions were found (*p* < 0.05): R152a generated the largest responses with PBT and VINYL (~16 nA and ~5.6 nA, respectively), R227ea showed higher responses with POM and LDPE (~8 nA), and R134a delivered the highest flow rates across materials but exhibited limited electrical responsiveness. **Conclusions:** Ethanol addition mitigates undesirable electrostatic effects in HFC-based propellants by promoting charge dissipation. The results demonstrate the strong material dependence of triboelectric behaviour and underline the importance of optimising propellant–polymer pairings to minimise the electrostatic adhesion of aerosolised particles and improve pMDI drug delivery performance.

## 1. Introduction

Triboelectrification, the generation of electrostatic charge through frictional or interfacial interactions, is a well-characterised phenomenon in solid materials [1]. While the electrostatic behaviour of solid materials has been widely investigated, the electrostatic phenomena in pressurised metered-dose inhalers (pMDIs) remain comparatively underexplored, despite the long-standing recognition of their impact on the efficiency and reproducibility of pulmonary drug delivery [2]. In pMDIs, both triboelectric charging and electrokinetic effects can arise as the propellant mixture undergoes high-velocity flow and interacts with the internal surfaces of the inhaler [3]. These conditions promote charge separation at the liquid–wall interface and within the aerosolised spray, driven by shear forces and phase transitions. However, despite advances in device engineering and formulation science [4], the complex coupling between charge dynamics, aerosol transport, and intrapulmonary drug dispersion has not yet been accurately modelled [5,6].

Most commercially available pMDIs incorporate polymer components whose inner surfaces are microscopically rough and partially amorphous. These surface irregularities introduce local variations in work function by facilitating charge trapping and molecular adsorption, which can influence dose reproducibility through altered aerosol deposition and enhanced triboelectric generation. Current strategies to mitigate these electrostatic effects primarily involve material modification, employing low-friction, low-surface-energy polymers such as POM (polyoxymethylene; –(CH_2_–O)–) or applying antistatic coatings, including graphene-based conductive films or conductive additives to promote charge dissipation. However, these approaches present practical challenges for large-scale manufacturing, as coatings often fail to maintain uniform smoothness and can substantially increase production costs [7,8].

Recent studies emphasise the need to reassess aerosol electrostatics in the context of formulation and device design [9]. Among the device’s components, the spacer exerts a strong influence on electrostatic interactions. Charged particles can adhere to the inner surfaces of the spacer, leading to drug loss, or agglomerate into larger clusters with reduced mobility [10,11]. This enhances deposition on the mucosal surfaces of the mouth, throat, and upper airways, further diminishing the fraction of drug reaching the lower respiratory tract (alveolar region of the lungs) [12,13].

In addition to the device’s configuration, formulation components also impact electrostatic behaviour [5]. Ethanol (CH_3_CH_2_OH) is commonly employed as a co-solvent in pMDIs to enhance drug solubility and optimise aerosol characteristics [14]. As a polar solvent, ethanol can also influence the electrostatic properties of propellant systems. However, its impact on electrostatic charge behaviour has not been extensively characterised, particularly in mixtures of 1,1-difluoroethane (HFA-152a; CH_3_CHF_2_) and ethanol. Ethanol possesses a relatively high dielectric constant (ε≈25) compared to hydrofluorocarbons (HFCs) and hydrofluoroalkanes (HFAs) (ε<12) [15] and is hypothesised to facilitate charge dissipation by enhancing charge mobility and promoting charge redistribution within the liquid phase [16]. This may reduce localised charge accumulation on aerosolised drug particles, thereby minimising electrostatic adhesion to spacer surfaces and mitigating particle agglomeration. Furthermore, ethanol’s high miscibility with HFA/HFC propellants and its ability to lower both viscosity and surface tension contribute to improved droplet formation and aerosol dispersion, making it particularly advantageous in pMDI formulations [17]. As observed in electrostatic studies of dielectric fluids, increased formulation conductivity shortens the charge relaxation time, thereby reducing the electrostatic charge carried by the aerosol as it exits the inhaler and enters the lungs [18].

To investigate the triboelectric and electrokinetic charging mechanism, an experimental setup consisting of a tube connected to the output of a canister dispensing the propellant/propellant mixture at controlled flow rates was employed. The resulting current was measured across the ends of the tube using a high-sensitivity electrometer. In addition to the effects of flow velocity, propellant, and concentration of ethanol, the tube material was found to significantly influence charge behaviour [19]. To systematically examine material–propellant interactions, three propellants, R134a (1,1,1,2-tetrafluoroethane, C_2_H_2_F_4_), R227ea (1,1,1,2,3,3,3-heptafluoropropane, C_3_HF_7_), and R152a (1,1-difluoroethane, CH_3_CHF_2_) were tested with four tubing materials: low-density polyethylene (LDPE, –(CH_2_–CH_2_)–), polybutylene terephthalate (PBT), polyoxymethylene (POM), and vinyl (polyvinyl chloride, PVC; –(CH_2_–CHCl)–). Among these combinations, the LDPE-R152a pair showed the least current variability and was therefore selected for further experiments investigating the electrostatic behaviour of propellant–ethanol mixtures.

According to the triboelectric series, a ranked list of materials based on their tendency to gain or lose electrons, materials such as low-density polyethylene (LDPE) are positioned toward the positive end, indicating a propensity to donate electrons and become positively charged upon contact. LDPE, due to its electropositive character, tends to lose electrons when in frictional contact with most other materials, including those present in aerosol formulations [20,21].

As the high-velocity, low-conductivity 152a–ethanol mixture flows through the LDPE tube, intense shear forces and surface interactions enhance the generation of triboelectric charge. This effect is further amplified by continuous surface contact under dynamic flow conditions [6]. Thus, the selection of LDPE in the experimental configuration directly influences both the polarity and magnitude of the resulting electrostatic charges. Its low surface energy and electron-donating characteristics contribute to the elevated current levels observed [22,23]. A detailed explanation of the current generation mechanism and associated analysis is provided later in this manuscript, supported by mathematical modelling.

This study highlights the critical influence of ethanol on the triboelectric charging behaviour of 152a-based pMDI aerosols. The experiments demonstrated a clear, approximately linear increase in triboelectric current with rising ethanol concentration. These findings add to the growing evidence supporting the inclusion of electrostatic considerations in the design and regulation of inhalation therapies. Strategically modulating triboelectric properties, guided by empirical data and supported by physical theory, offers a promising approach for enhancing the efficiency, reliability, and consistency of drug delivery in respiratory medicine [24].

## 2. Results and Discussion

**Figure 1:**
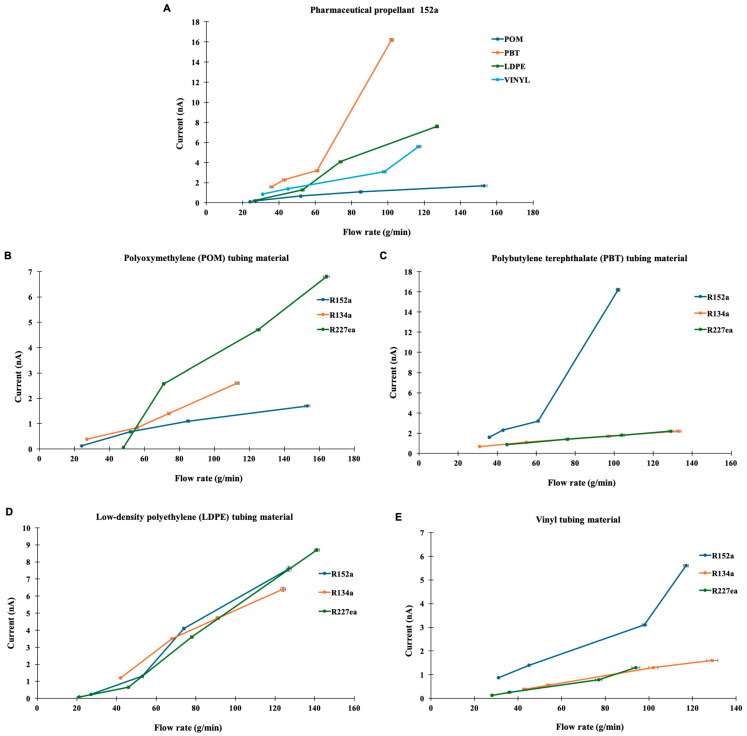
Current response as a function of flow rate for various propellants and tube materials. (**A**) Current (amps) as a function of flow rate (g/min) for R152a propellant through different tube materials. (**B**) Flow rate dependence of current for propellants R152a, R227ea, and R134a through a polyoxymethylene (POM) tube material. (**C**) Flow rate dependence of current for propellants R152a, R227ea, and R134a through a polybutylene terephthalate (PBT) tube material. (**D**) Flow rate dependence of current for propellants R152a, R227ea, and R134a through a low-density polyethylene (LDPE) tube material. (**E**) Flow rate dependence of current for propellants R152a, R227ea, and R134a through a vinyl tube material. Representative error bars (±1–2% on both X and Y axes) are shown for visual clarity. Measurement variability in current was minimal across repeated runs, while flow-rate fluctuations contributed slightly to horizontal uncertainty.

Preliminary experiments were carried out to evaluate the effect of propellant type and tubing material on electrostatic current generation. Tests were performed using three propellants, R134a, R227ea, and R152a across four tubing materials: LDPE, PBT, POM, and vinyl. The measured currents were consistently below 10 nA, as illustrated in Figure 1.

Figure 1A shows the results for R152a flowing through each tubing material. PBT exhibited the highest current, indicating a strong electrostatic interaction between the polymer surface and the flowing propellant. This finding aligns with previous reports suggesting that materials with higher dielectric constant and lower conductivity, such as PBT, tend to accumulate greater charge densities due to reduced change dissipation along the surface [25]. LDPE showed a moderate current that increased linearly with flow rate, consistent with earlier observations that polyethylene materials generate charges primarily through surface contact and separation rather than bulk polarisation [26]. Vinyl and POM displayed relatively low and stable current profiles, which may result from their higher surface conductivity and smoother surface morphology that facilitate faster charge relaxation.

The general trend broadly aligns with the relative behaviour of the materials in the triboelectric series (PBT > POM > LDPE > vinyl) [27]. Although the series was originally derived for dry solid–solid contacts, it can be qualitatively extended to liquid–solid systems, since the same liquid interacts with each material under identical flow conditions. Interestingly, despite POM’s relatively higher position in the series, it generates a lower current than expected. This reduced charge generation may stem from POM’s low coefficient of friction and high abrasion resistance [28], which limit interfacial shear and the real contact events necessary for effective charge transfer.

Since R152a produced measurable responses across all tubing types, Figure 1B compares all three propellants flowing through POM tubing. Only R227ea exhibited a noticeable increase in current with flow rate, whereas R134a and R152a showed relatively flat responses. These differences can be attributed to variations in molecular polarity and vapour pressure among the propellants. Similar trends have been noted by Vervaet and Byron (1999) [29], who reported that lower molecular weight and more polar fluorocarbons propellants exhibit stronger interactions with formulation components and container surfaces, which could enhance intermolecular collisions and facilitate charge transfer processes. These differences in current response among R134a, R227ea, and R152a likely arise from their distinct dielectric constant and electrical conductivities, as previously reported for similar refrigerant systems [30].

Figure 1C presents the results from PBT tubing, where R152a generated significantly higher currents compared to R134a and R227ea, both of which produced minimal currents. This enhanced response is likely related to the higher dipole moment of R152a, which promotes charge separation upon interaction with polar polymer surfaces, consistent with observations that polymer surface polarity strongly governs triboelectric charge transfer [31]. In contrast, R134a and R227ea were less polar, exhibiting weaker charge transfer efficiency under similar conditions.

In Figure 1D, LDPE tubing exhibited a substantial and linear increase in current for all three propellants. At lower flow rates (<70 g/min), R134a produced the highest current, while R152a and R227ea generated higher currents at elevated flow rates (>70 g/min). The linear trend supports the notion that flow velocity strongly influences triboelectric charge generation by increasing the frequency of fluid–wall collisions, as also discussed in recent analyses of polymer-based triboelectric systems [32]. The relatively stable yet progressively increasing current observed for LDPE is consistent with its semi-crystalline structure and moderate dielectric constant, which facilitate gradual charge accumulation under flow [33].

Figure 1E shows the performance of vinyl tubing with all three propellants. R152a again produced the most pronounced response, with a sharp rise in current above 100 g/min, whereas R134a and R227ea remained consistently low. The strong dependence of current on flow rate and propellant type indicates that both material properties (dielectric constant, conductivity, and surface energy) and fluid characteristics (polarity and molecular weight) govern triboelectric behaviour in such systems. Overall, the results indicate a generally linear relationship between flow rate and current across all material–propellant combinations, confirming that fluid velocity is a key factor in electrostatic charge generation within the system. Moreover, the observed material-dependant trends agree with previous studies of tribocharging in polymer–gas systems, which emphasise the critical role of interfacial electronic interactions and dielectric mismatch between the contacting phases [34,35].

Vertical variability in current measurements was minimal across repeated runs, whereas higher variability in flow rate resulted from valve-control fluctuations. Error bars were therefore originally excluded, as they would primarily represent flow-rate uncertainty rather than current instability (Figure 1). In the revised version, representative error bars of ±1–2% were applied to both axes to illustrate measurement uncertainty. This range reflects the negligible vertical variability in the current and the small flow-rate fluctuations observed during repeated tests.

Table 1 further indicates that the LDPE-152a combination demonstrates the lowest variance among all tested configurations. In a previous study [19], R152a was shown to exhibit significantly lower resistivity compared with R134a and 227ea. Furthermore, the R152a–ethanol mixture in LDPE tubing maintained the lowest resistivity up to a certain ethanol concentration. These findings, together with the current observation of low measurement variability, support the selection of the LDPE-R152a system for subsequent experiments. This configuration was therefore chosen to investigate current generation as a function of ethanol concentration.

**Figure 2:**
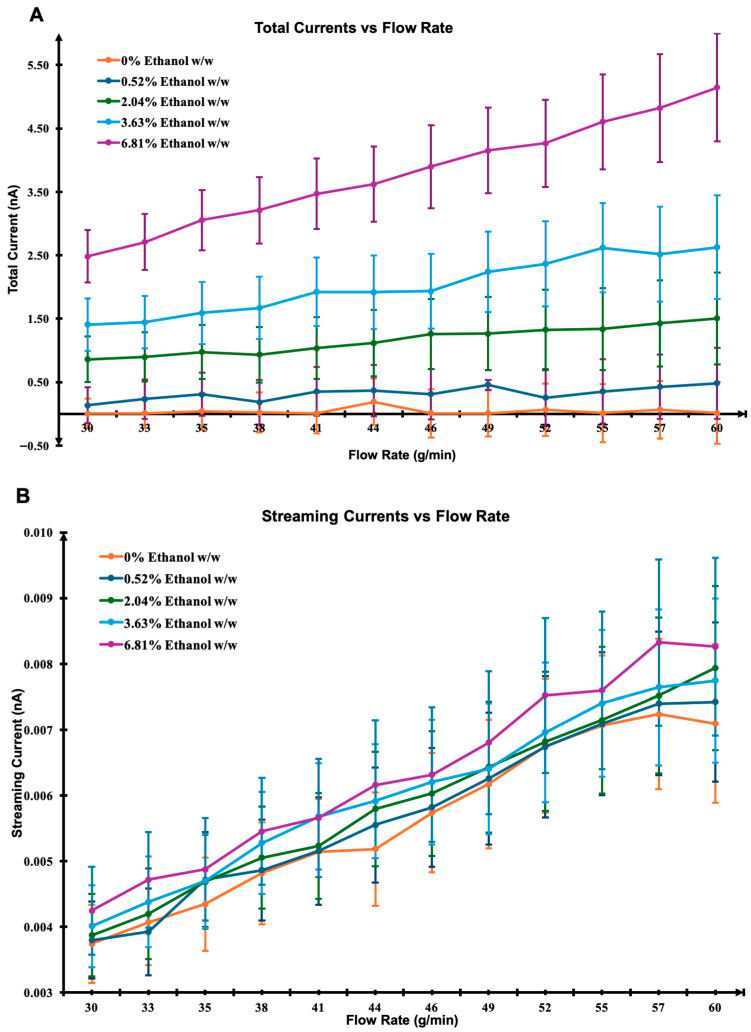

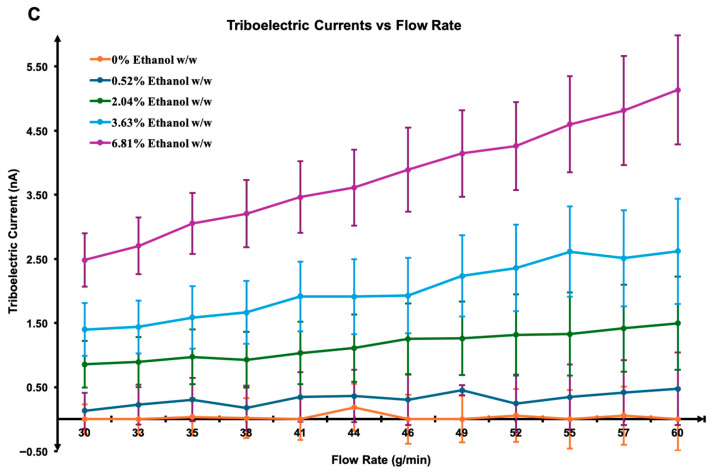
Simulated current responses as a function of flow rate and ethanol concentration. (**A**) Total current, (**B**) streaming current based on the Helmholtz–Smoluchowski model, and (**C**) empirically modelled triboelectric current. The simulations illustrate the relative contributions and trends of each current component under varying conditions.

Simulations were conducted under several simplifying assumptions, including fully developed laminar Poiseuille flow, a constant zeta potential, the neglect of charge recombination, axial diffusion, and space charge effects. Total current was calculated across various flow rates and ethanol concentrations (Figure 2A), capturing the contributions from both streaming and triboelectric currents.

Fluid properties such as density, viscosity, and dielectric constant were determined using mixture rules based on the weight-to-weight ratio of ethanol and R152a. The streaming current (Figure 2B) was estimated using the Helmholtz–Smoluchowski equation, incorporating parameters such as dielectric permittivity, viscosity, and the applied pressure gradient. This theoretical framework is consistent with prior electrokinetic models for dielectric fluids, where charge displacement along the interface is governed by the interplay between viscous drag and electric double layer potential [36,37]. In contrast, the triboelectric current (Figure 2C) was empirically modelled as a function of average axial velocity and wetted surface area, with the triboelectric coefficient (α) assumed to scale linearly with ethanol concentration. Similar empirical treatments have been applied in modelling charge generation in multiphase and aerosol systems, where α represents the material-dependent efficiency of charge transfer during interfacial collisions [31,34]. Results show that the streaming current increases linearly with ethanol concentration, a trend partly driven by the reduced viscosity of ethanol-rich mixtures. This behaviour agrees with the predictions of the Helmholtz–Smoluchowski theory, in which increased permittivity or decreased viscosity amplifies electrokinetic charge transport [32]. While triboelectric current also rises with increasing ethanol concentration, the relationship appears non-linear. Such non-linearity is consistent with previous reports suggesting that material-dependant factors including molecular polarity and hydrogen-bonding capability can strongly influence charge transfer efficiency at dielectric interfaces [38], particularly under dynamically varying or non-equilibrium conditions. Overall, the model supports the hypothesis that the streaming current contribution is significantly smaller than the triboelectric current under these conditions. This is in line with experimental studies on dielectric liquid flows, where charge separation due to contact electrification dominates over electrokinetic streaming effects at moderate flow rates [26,34]. The agreement between simulation trends and theoretical expectations thus reinforces the central role of triboelectrification as the primary charge generation mechanism in ethanol–HFC mixtures.

Additionally, Table 2 presents the measured current as a function of flow rate for R152a across various valve stem materials. The data further emphasise the role of material selection in triboelectric charging, showing that the valve stem composition can significantly influence the magnitude and behaviour of current generation under dynamic flow conditions.

**Figure 3:**
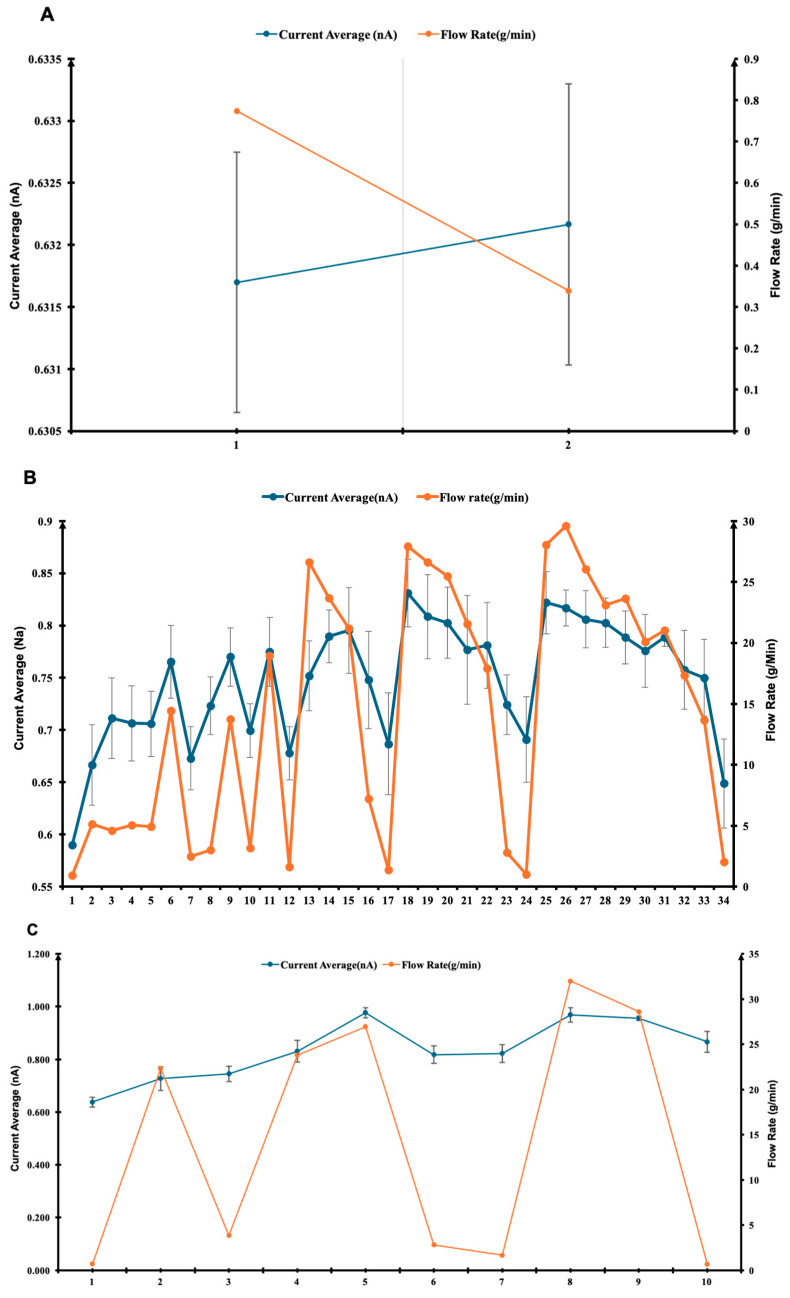

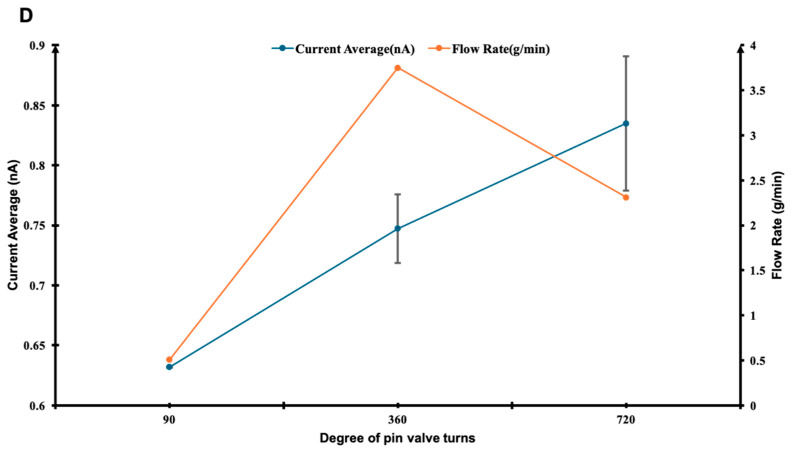
Time-resolved measurements of current and flow rate fluctuations for R152a under different pin valve settings: (**A**) quarter rotation (90°), (**B**) full rotation (360°), and (**C**) two full rotations (720°). (**D**) Summary plot comparing the stability and responsiveness of each configuration, showing the effect of pin valve opening on average current and flow rate. The *x*-axis represents the degree of pin valve turns, the left *y*-axis shows the average current (nA), and the right *y*-axis indicates the corresponding flow rate (g/min). (**A**–**C**) *x*-axis represents the number of repeated measurements.

Figure 3 illustrates how the measured current responds to changes in flow rate and demonstrates the system’s stability across all of the tested conditions. In Figure 3A, a low-resolution test was conducted with a quarter valve opening (90°). The flow rate gradually decreased over time, while the current exhibited a slight increase. This behaviour may be attributed to localised turbulence and increased shear stress near the partially constricted valve, which enhance fluid–surface interactions and promote charge separation even under reduced mass flow conditions. Similar effects have been reported in studies of flow-induced electrification, where micro-vortices and transient eddies amplify interfacial charge transfer by increasing the contact frequency between liquid molecules and dielectric walls [34,37].

Figure 3B presents a higher-resolution test with a full valve rotation (360°). Although both current and flow rate show fluctuations, their trends remain closely correlated, highlighting the system’s sensitivity to flow dynamics. The observed current–flow coupling is consistent with triboelectric charge generation models, which predict a quasi-linear relationship between wall shear rate and charge density under laminar-to-transitional flow regimes [26,32]. The minor oscillations may result from transient flow pulsations or microbubble entrainment, both of which have been shown to influence the instantaneous current in dielectric fluids [25].

In Figure 3C, the valve was opened to 720° (two full rotations). Here, the current remains relatively stable, while the flow rate shows larger but still controlled fluctuations. This indicates that, beyond a certain threshold, additional increases in flow velocity produce diminishing returns in charge output, likely due to partial charge neutralisation or saturation effects at the polymer surface [31]. The result aligns with prior findings that excessive turbulence can reduce net charge transfer by enhancing charge recombination in the bulk fluid [35].

Figure 3D summarises the average current and flow rates across the three valve settings. The current increases modestly with greater valve opening, whereas the flow rate peaks at 360° and decreases at 720°. This non-monotonic behaviour may result from complex interplay between hydrodynamic turbulence and interfacial charge saturation. Similar non-linear dependencies between flow velocity and current have been reported in polymer–fluid triboelectric systems, where both mechanical and electrical relaxation timescales influence charge accumulation [29,34]. Together, these results confirm that the apparatus provides reliable current measurements and responds effectively to variations in flow rate. Minor flow irregularities are likely due to external disturbances such as vibration, temperature fluctuations, or electronic noise, which have been identified as secondary noise sources in flow electrification studies [37]. Based on the observed stability and responsiveness at the 360° valve setting, all experiments described in this paper were conducted under this configuration. The reproducibility and controlled response across valve openings validate the system’s suitability for investigating triboelectric effects under dynamic flow conditions.

**Figure 4:**
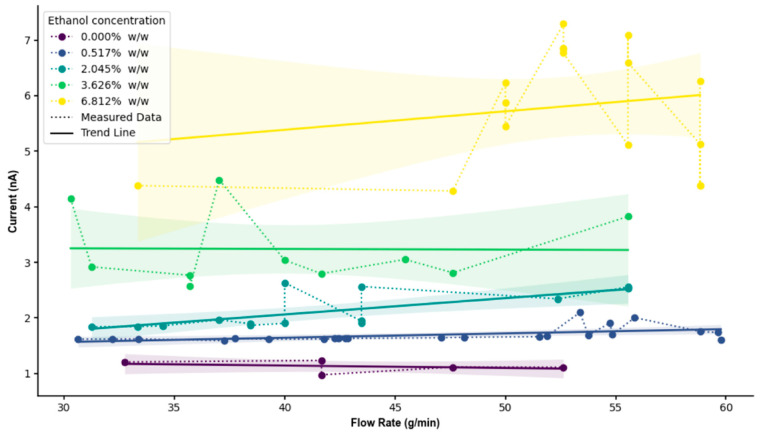
Measured current and flow rate data from the experiment plotted with different ethanol concentrations. The shaded region represents the 95% confidence interval of the fitted mean current-flow relationship, indicating the range within which the true trend line is expected to lie with 95% confidence.

Experimental results shown in Figure 4, using an R152a/ethanol mixture with LDPE tubing, reveal a clear linear increase in current with flow rate across all ethanol concentrations. At low concentrations (0% to 2.045%), the current remains low (~1.7 nA) and relatively flat across flow rates, indicating minimal charge mobility and separation. This behaviour is consistent with the low polarity and dielectric constant of pure HFC-152a, which limits both charge density and interfacial charge transfer [34,35]. At a mid-range concentration (3.626%), the current increases moderately (~3–4 nA), suggesting a transitional regime where ethanol begins to modify fluid permittivity and surface charge exchange. Similar non-linear transitions have been reported in binary liquid mixtures, where polar additives alter double-layer dynamics and charge transport through changes in viscosity and permittivity [37,39]. The relatively flat current–flow relationship within this regime may indicate a balance between increased dielectric constant and enhanced energy dissipation through intermolecular collisions. This behaviour may also be explained through modification in the electric double-layer structure at the LDPE interface, as increased ethanol content alters dielectric screening and charge relaxation dynamics [40].

At a high ethanol concentration (6.812%), the current increases substantially (up to ~7 nA) with a pronounced positive slope, indicating that ethanol dominates the fluid’s electrostatic behaviour. The deviation from perfect linearity at these higher concentrations may also reflect local compositional heterogeneity and micro-phase interactions within the R152a–ethanol mixture, which can influence charge distribution and interfacial polarisation [41]. Ethanol’s high dielectric constant facilitates enhanced charge separation and migration along the tube’s surface [25,31]. Comparable findings were observed by Meng et al. (2025), who demonstrated that polymer–fluid charge transfer efficiency increases with solvent polarity and interfacial mobility [32]. Overall, the rise in current magnitude with increasing ethanol content is attributed to ethanol’s ability to promote charge dissipation and modify surface states, thus amplifying triboelectric exchange. The observed linearity between current and flow rate is consistent with the dominance of the velocity-driven charge generation mechanism described in previous electrohydrodynamic studies [37,39].

While the overall experimental trend agrees with the simulation, greater variability at high ethanol concentration (6.812%) likely arises from multiple physical factors. At these concentrations, the mixture no longer behaves as a single homogeneous dielectric medium but rather as a dynamic micro-segregated two-phase flow [41]. This can generate transient mixing zones within the stream, consistent with the increased current fluctuations observed as ethanol concentration rises. Ethanol-rich and R152a-rich domains along the LDPE wall may lead to localised charge accumulation and rapid current spikes. Additionally, ethanol’s interaction with polymer surface, including softening or swelling, can modify the local contact angle and the effective wetted area, further contributing to current instability [42,43]. Similar experimental scatter has also been noted in multiphase triboelectric systems due to charge relaxation asymmetry and turbulence-induced intermittency [44,45]. Notably, these fluctuations predominantly occur after some flow time rather than at initiation, suggesting that gradual changes in the liquid–solid interface function as the surface becomes increasingly polarised by ethanol absorption [7].

Thus, both the simulation and experiment demonstrate strong qualitative alignment, confirming that increasing ethanol concentrations and flow rates enhance triboelectric current generation. The mathematical model effectively captures the linear current–velocity dependence and the dominant electrokinetic–triboelectric coupling, validating its underlying assumptions. Minor discrepancies likely arise from local zeta potential variation or interfacial heterogeneity at high ethanol levels, phenomena that warrant future exploration through transient or molecular-scale modelling.

**Figure 5:**
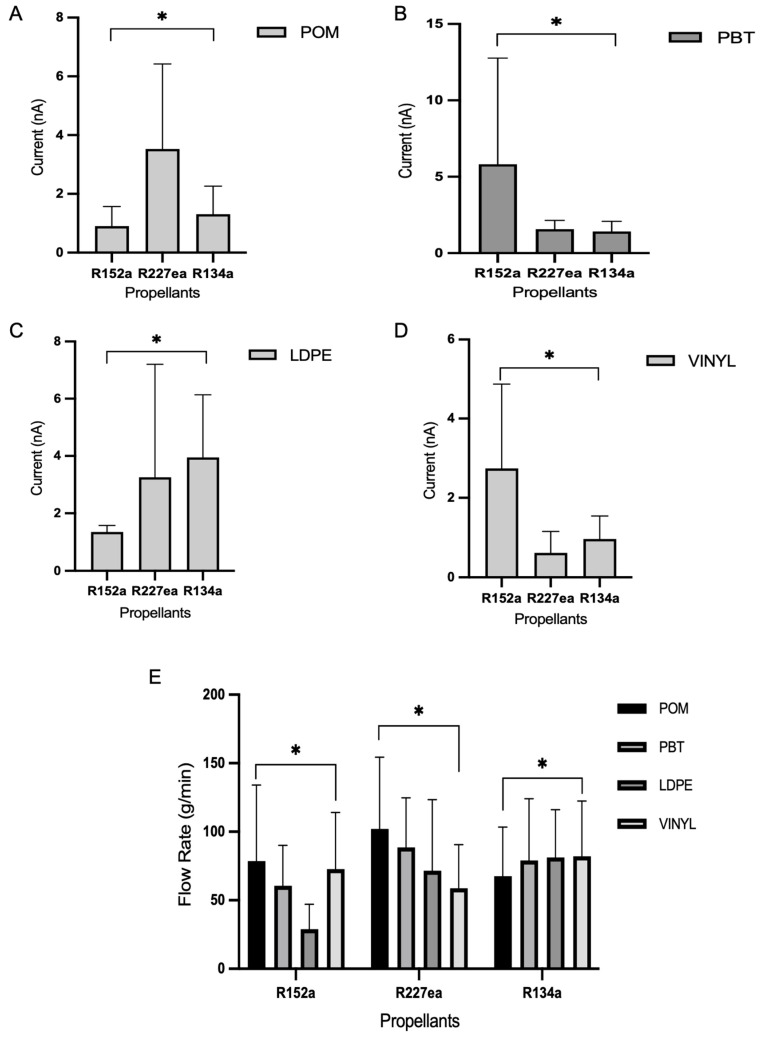
Statistical comparison of current outputs across different propellants for each material, based on the current-related data shown in (**A**–**D**). (**E**) Presents flow rate comparisons across materials and propellants and is included in the overall analysis as a separate dependent variable. Data were analysed using two-way ANOVA followed by Tukey’s multiple comparisons test. Asterisks (*) indicate statistically significant differences (*p* < 0.05) between propellants within each material group.

Figure 5 presents the statistical analysis comparing propellant effects across materials. The presence of statistically significant differences (*p* < 0.05) reinforces the trends reported in Figure 2, Figure 3, Figure 4 and Figure 5 and confirms that both propellant identity and tubing material are critical determinants of current output. In several comparisons, the analysis also revealed significant propellant–material interactions (*p* < 0.05), indicating non-additive effects where the influence of a given propellant depends on the polymer substrate.

In Figure 5A (POM), currents are moderate for all propellants, ranging from approximately 2 to 8 nA. Among them, R227ea produced the highest values, followed by R134a and R152a. The statistical significance of these group differences (*p* < 0.05) suggests that intrinsic fluid properties such as resistivity and molecular structure (here, R227ea’s relatively high resistivity of 1.31 × 10^10^ Ω·m) modulate charge generation when paired with the POM surface. This behaviour is consistent with studies showing that fluid resistivity and interfacial electronic properties govern the efficiency of charge transfer in liquid–solid systems [29,34].

Figure 5B (PBT) shows a markedly different pattern: R152a yields a peak current of approximately 16 nA, while R227ea and R134a remain below 3 nA (*p* < 0.05). The strong, material-dependent response of R152a implies favourable matching between the propellant’s molecular polarity/interaction potential and the chemical or physical surface properties of PBT (surface energy, functional groups, or local dielectric environment). Recent reviews and experimental work on polymer interface engineering support this mechanism, showing that surface chemistry and dielectric mismatch strongly influence triboelectric charge transfer efficiency [25,31,32].

For LDPE, (Figure 5C), currents are clustered (≈1.5–8 nA) and differences remain statistically significant (*p* < 0.05) though less pronounced than with PBT. This suggests LDPE’s lower surface polarity and higher ability to dissipate charge moderate propellant-dependent effects, again aligning with prior observations that neutral, low-polarity polymers tend to be less responsive to variations in fluid chemistry [34,35].

In Figure 5D (vinyl) mirrors PBT’s outcome: R152a produces the highest current (~5.6 nA), with R227ea and R134a significantly lower (*p* < 0.05). The repeated strong performance of R152a on PBT and vinyl supports the interpretation that R152a’s molecular properties (polarity, dipole moment, or interaction propensity) couple efficiently to certain polymer surfaces to enhance charge exchange. This material-selective behaviour is well documented in the triboelectric literature and reinforces the importance of interface chemistry for device optimisation [25,31].

Figure 5E summarises flow rates (g/min) across materials: R134a consistently attains the highest flow rates, followed by R227ea, then R152a (all differences *p* < 0.05). The higher flow of R134a likely reflects its volatility and lower viscosity, yet high flow did not uniformly translate to higher current (e.g., PBT and vinyl). This decoupling, where flow rate and electrical output are not positively correlated, indicated that hydrodynamic factors (residence time and shear) alone cannot explain electrification; interfacial compatibility and material electrical properties are decisive [32,37].

Taken together, the statistical outcomes clarify a complex interplay between the fluid’s electrical/physical properties and polymer surface characteristics. R227ea performs best with POM and LDPE, whereas R152a is most effective with PBT and vinyl; R134a achieves the highest flow but limited electrical effectiveness in several materials. These patterns emphasise that optimal propellant–material parings are application specific and must balance both flow dynamics and triboelectric compatibility [29,31].

## 3. Materials and Methods

### 3.1. Materials

The study aims to investigate the triboelectrification and electrokinetic effects arising from interactions between propellants and tube materials. Commonly used propellants and tube materials in pMDIs were selected for the study. Two hydrofluorocarbons (HFC-152a and HFC-134a) and one hydrofluoroalkane (HFA-227ea) were used to evaluate their electrostatic behaviour. All three propellants were provided by Zephex^®^, Orbia Fluor and Energy Materials, Mexichem UK Ltd., Chester, UK. Each propellant was passed through tubing made from LDPE (Adtech Polymer Engineering Ltd., Gloucestershire, UK), PBT (SOG Ltd., Runcorn, UK), POM (SOG Ltd., Runcorn, UK), and vinyl (PVC) (Adtech Polymer Engineering Ltd., Gloucestershire, UK) to analyse electrostatic charge accumulation in both the fluid and the surface [17]. Electrostatic charging is highly sensitive to the physicochemical properties of both the propellant and the tubing surface.

The relative permittivity of the propellant influences both the extent of charge separation and the localization of the charges at the surface. Among the propellants studied, 152a has the lowest relative permittivity and 227ea has the highest. As a result, charge dissipation is expected to be greater in 227ea compared to the HFCs. The density and the viscosity of the propellants affect the shear forces during flow, which in turn impact the degree of charge separation. On the other hand, the dielectric constant of the tube material influences the rate at which accumulated chargers dissipate through the surface. LDPE, being highly insulating and tribo-negative, tends to favour more charge accumulation compared to the rest of the tube materials [46]. Throughout this experiment, various combinations of tubing materials, propellants, and mixed ethanol concentrations with propellants were tested (Table 3).

### 3.2. Methods

#### 3.2.1. Flow-Through Cell

**Figure 6:**
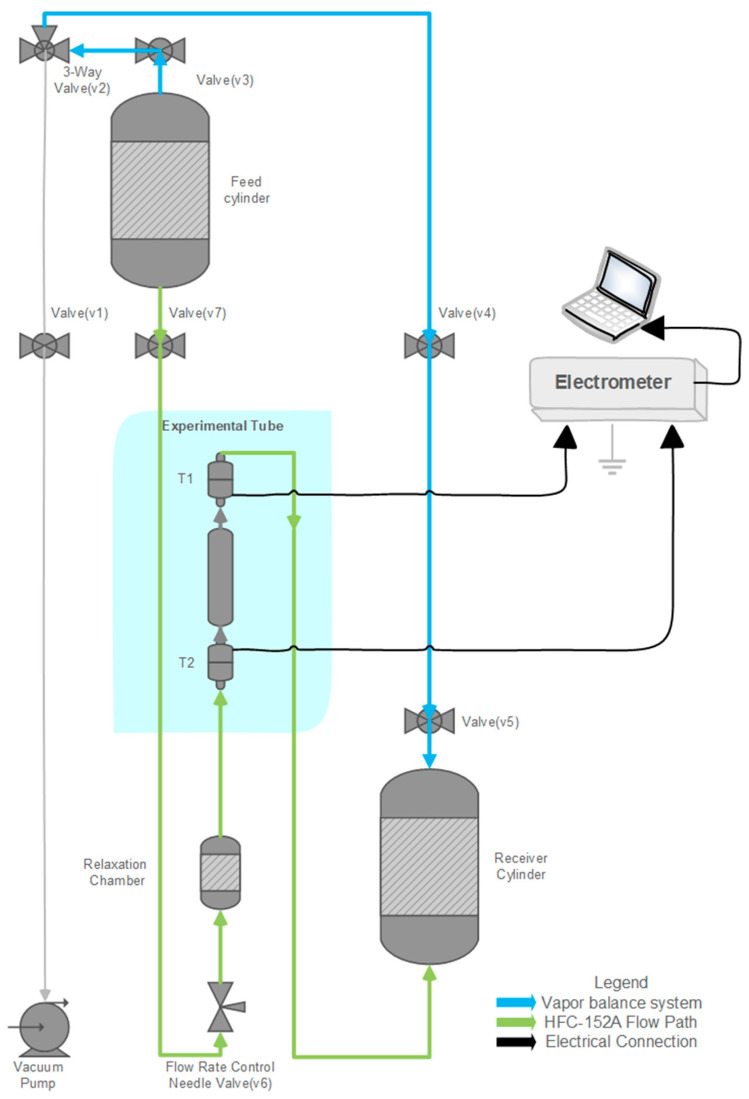
Schematic diagram of the experimental setup illustrating the flow path of the propellant from the feed cylinder through the relaxation chamber and test section to the receiving cylinder. Key components include the needle valve for flow control and electrodes for current measurement.

A custom test rig was designed to pass propellants through various tubing materials, simulating the mechanism of pMDIs under controlled conditions and at reduced flow speeds. The setup comprised two primary stainless-steel cylinders: a feed cylinder and a receiving cylinder, as shown in Figure 6. An experimental tube was positioned between the cylinders, with an intermediate relaxation chamber to allow the propellant to reach pressure and temperature equilibrium before entering the test tube.

The entire system can be evacuated using a vacuum pump connected via dedicated tubing to remove any residual gasses before each test. The feed cylinder is mounted at the top of the fixture, while the receiving cylinder is positioned on a precision weighing scale. The change in collected mass over time is used to calculate the flow rate (g/min).

To measure the electrostatic charge generated through triboelectrification and electrokinetic processes, a Keithley 6517B electrometer (Keithley Instruments, Solon, OH, USA) was connected across both ends of the experimental tube. A custom Laboratory Virtual Instrument Engineering Workbench (LabVIEW-2023, Q1, National Instruments, Austin, TX, USA) programme was developed to record and monitor the real-time current produced during propellant flow.

#### 3.2.2. Preparation of Samples

The feed cylinder, with a total internal volume of 500 mL and a defined safe working volume of 400 mL, was used to prepare and store propellant samples for testing. To ensure safe operation across all propellants of varying density, a mass limit of 350 ± 5 g was imposed. This limit was chosen to prevent overfilling, particularly when using higher-density propellants such as R227ea.

Propellants were transferred from the stationary source cylinder into the evacuated feed cylinder until total mass reached approximately 350 g, remaining within the safe margin. Following the transfer, the cylinder was allowed to equilibrate at room temperature for 30–60 min, to permit phase and pressure stabilisation before testing.

For formulations containing ethanol, the ethanol mass was pre-calculated based on the target concentration (*w*/*w*). The feed cylinder was first evacuated to remove any residual substances. A pre-measured amount of dried ethanol (absolute ethanol, assay 99.88%, VWR Chemicals, Lutterworth, Leicestershire, UK), previously dried at Orbia Flour and Energy Materials using a Honeywell/UOP 3A molecular sieve (Sigma Aldrich, Gillingham, Dorset, UK), was then injected. The actual mass of ethanol added was determined by weighing the cylinder before and after injection. Ethanol was added first to prevent premature vaporisation during subsequent propellant injections.

Following ethanol addition, the calculated amount of propellant was transferred into the cylinder, ensuring that the total mass did not to exceed 350 g. The final mass of the loaded cylinder was recorded, allowing for an accurate determination of the ethanol concentration in the mixture prior to each experiment.

#### 3.2.3. Performing the Flow-Through Cell Tests

Prior to initiating the experiment, the entire system, excluding the feed cylinder, was evacuated to remove any residues or contaminants that could contribute to unexpected triboelectric charging. Additionally, all components were grounded to eliminate any pre-existing electrostatic charges within the system.

Once the system and the feed cylinder containing the sample were sealed and securely fixed, the valves were opened to initiate flow from the feed cylinder to the receiving cylinder through the tube under test. Simultaneously, data acquisition commenced: the mass increase in the receiving cylinder was continuously recorded by a precision scale, while the electrometer logged the triboelectric current generated across electrodes every 100 ms. Current data were acquired and timestamped using a LabVIEW virtual instrument.

After data acquisition, the mass and current streams were time-aligned and analysed to extract instantaneous flow rates (calculated as the time derivative of the mass curve) and the corresponding electric current. This allowed for a quantitative comparison of electrostatic charging behaviour across different propellants and propellant mixtures. Before testing the propellants, pure R152a was used to evaluate the stability and responsiveness of the experimental setup and measurement system. The flow rate and current were measured simultaneously over extended periods.

To modulate the flow, the needle valve located upstream of the relaxation chamber was set to three discrete positions: quarter rotation (90°), full rotation (360°), and two full rotations (720°).

After confirming the stability and responsiveness of the setup, tests were conducted using three propellants: R134a (Zephex^®^, Orbia Fluor and Energy Materials, Mexichem UK Ltd., Chester, UK), R227ea (Zephex^®^, Orbia), and R152a (Zephex^®^, Orbia), in combination with four tubing materials: LDPE, vinyl (Adtech Polymer Engineering Ltd., Gloucestershire, UK), POM, and PBT (SOG Ltd., Runcorn, UK). The resulting current measurements were statistically analysed to identify the most stable material–propellant combination, which was subsequently selected for experiments with ethanol mixtures.

#### 3.2.4. Simulation Method

Simulation models were developed using Python 3.13.1 (Python Software Foundation, Wilmington, DE, USA) to quantitatively evaluate electrostatic currents generated during the flow of R152a–ethanol mixtures through LDPE tubing. A custom Python script was created to calculate and visualise the predicted current as a function of flow rate and ethanol concentration, based on the governing equations described in the Methods section. Two current-generation mechanisms were modelled: (a) a streaming current, calculated using the Helmholtz–Smoluchowski equation, and (b) a triboelectric current, modelled empirically using a triboelectric coefficient, assumed to vary linearly with ethanol concentration and scale proportionally with flow rate.

Fluid properties, including density, viscosity, and relative permittivity were dynamically calculated based on ethanol concentration. Mixture rules were applied, assuming ideal volume additivity for density, while viscosity and permittivity were computed as weighted averages of the pure components. These computed values were used as inputs for the current models.

Simulation outputs included predicted total current as a function of flow rate and ethanol concentration, with individual contributions from streaming and triboelectric mechanisms distinguished separately. Results were plotted using Matplotlib (Version 3.10.7; NumFOCUS Foundation, Austin, TX, USA) and all numerical computations were performed with NumPy. This framework enabled a direct comparison between predicted and experimental current values, supporting validation of the combined electrokinetic–triboelectric model.

##### Simulation of Current from 152a + Ethanol Mixture Through LDPE

The total current generated when a 152a and ethanol mixture flows through an LDPE tube under laminar conditions arises from three distinct mechanisms: a streaming current from electrokinetic flow (governed by the Helmholtz–Smoluchowski equation) [47,48], a triboelectric (frictional) current from direct fluid–solid contact charging [1,49], and a shear-induced current due to internal fluid velocity gradients.

$$I_{\mathrm{streaming}}=-\frac{\varepsilon_{r}\varepsilon_{0}\pi R^{2}}{\eta }\frac{\Delta P}{L}\zeta$$

where $\varepsilon_{r}$ is the relative permittivity of the fluid mixture (ethanol $\varepsilon_{r}\approx 24.6$ at 25  °C, 152a $\varepsilon_{r}\approx 6.0)$ $\varepsilon_{0}$ is the vacuum permittivity ($8.854\;\times 10^{-12}$ F/m), η is the dynamic viscosity (ethanol η≈1.2 mPa⋅s, 152a η≈0.13 mPa⋅s) [50,51], $\frac{\Delta P}{L}$ is the axial pressure gradient, and ζ is the zeta potential (ethanol estimated $\zeta \approx -\left(30\;-100\right)\;mV$ so approximating to be ζ≈−50 mV) [52]. A zeta potential range of −30 mV to –100 mV was used to reflect realistic variability at the LDPE–ethanol/R152a interface. The standard deviation in the triboelectrification coefficient was included to capture experimental variability. This range is supported by experimental measurements on polymer surfaces; Breite et al. (2016) report that electrolyte composition strongly affects the zeta potential of polymer membranes and Zanoni et al. (2025) measured LDPE surfaces exhibiting ζ≈−51 mV, consistent with the values used in our simulations [53,54].

The pressure gradient will be calculated using the Hagen–Poiseuille equation [55],

$$\mathrm{Pressure}\;\mathrm{gradient}\;(\Delta P/L)=\frac{8\eta v_{\mathrm{surface}}}{R^{2}}$$

The triboelectric current, representing wall frictional charging, is empirically related to surface velocity as

$$I_{\mathrm{triboelectric}}=\alpha v_{\mathrm{surface}}P(x)$$

where P(x) is the wetted perimeter per unit of the tube, defined as

$$P\left(x\right)=\frac{1}{L}\int_{0}^{L}A\left(x\right)dx$$

If the tube has a varying radius or width along its length, integrating *A*(*x*) over *x* yields the total surface area. Assuming a cylindrical geometry, the wetted perimeter simplifies to

$$P\;(x)=\;\frac{A_{\mathrm{surface}}}{L}=2\pi R$$

where R is the internal radius of the tube.

Thus, the triboelectric current can be expressed as

$$I_{\mathrm{triboelectric}}=\alpha v_{\mathrm{surface}}(\frac{A_{\mathrm{surface}}}{L})$$

where α is the fitted triboelectric coefficient (experimentally derived for a similar organic mixture) [21], $v_{surface}$ is the average flow velocity at the wall, and $A_{surface}$ is the total wetted surface area.

Internal shear within the flowing liquid also contributes a secondary, weaker component to the current; however, this shear current is negligible, as previous studies indicate that triboelectric charging primarily originates from wall shear rather than bulk fluid motion [56].

Therefore, total current generated from the flow can be represented as

$$I_{\mathrm{total}}=I_{\mathrm{streaming}}+I_{\mathrm{triboelectric}}$$

In this model, all physical parameters (viscosity, permittivity, and mixture density) are dynamically adjusted based on ethanol concentration to accurately capture the evolving fluid properties. Given the extremely low ionic conductivity of both 152a and its mixture with ethanol, the streaming current is expected to be substantially lower than the triboelectric current under typical experimental conditions [48,57]. The result of the mathematical modelling confirms this statement for this experiment.

#### 3.2.5. Statistical Analysis

Statistical analysis was conducted using GraphPad Prism (GraphPad Software 10.4.2, San Diego, CA, USA). A two-way ANOVA was used to evaluate the effects of propellant type and material type on the measured electrical current. For each material, current values were compared across the three propellants (R152a, R227ea, and R134a), and Tukey’s multiple comparisons test was applied for pairwise group comparisons.

Separately, a two-way ANOVA was conducted to analyse flow rate data across different materials and propellants, followed by Tukey’s test to identify significant differences between groups. In all analyses, differences were considered statistically significant at *p* < 0.05. Statistically significant comparisons are indicated by asterisks in the corresponding figures.

## 4. Conclusions

This study examined the influence of ethanol concentration and flow rate on the electrostatic behaviour of R152a–LDPE systems, representative of pharmaceutical aerosol propellants used in pressurised metered-dose inhalers (pMDIs). Increasing ethanol concentration enhanced the measured current, indicating improved charge mobility and reduced surface charge retention on LDPE. Ethanol appears to promote enhanced charge mobility, facilitating efficient charge dissipation. A modest but consistent increase in current with higher flow rates aligns with the velocity dependence of triboelectric charging. Theoretical modelling and statistical analysis (two-way ANOVA, *p* < 0.05) confirmed the combined effects of ethanol concentration and flow rate, supporting ethanol’s role as a key modulator of electrostatic behaviour in fluorocarbon propellants.

From a practical standpoint, the findings suggest that ethanol can be effectively employed to mitigate unwanted charge build-up and improve aerosol uniformity, provided that its concentration is optimised. Excessive ethanol, however, may introduce formulation and device challenges, such as phase separation during atomisation, material swelling or leaching from seals and elastomers, and, in rare cases, bronchospasm in sensitive patients. Furthermore, ethanol’s slower evaporation relative to propellants could influence dose reproducibility over time.

Experimental limitations include minor temperature fluctuations, the absence of active flow control in the gravity-fed setup, and flow rates lower than those of actual pMDI devices. Small, trapped air pockets during propellant transfer may also have contributed to pressure variability. Future work should incorporate active flow regulation, replicate clinically relevant spray conditions, and employ advanced surface characterisation techniques (e.g., XPS, AFM, and zeta potential) to deepen mechanistic understanding. Overall, these insights support the inclusion of electrostatic evaluation (triboelectric effect) in pMDI formulation and component design to ensure more stable, efficient, and predictable drug delivery performance.

## Figures and Tables

**Table 1:** Variance in current measurements obtained from different combinations of propellants and tubing materials. The results were used to identify the most stable configuration for subsequent ethanol testing.

Variance	R134a	R152a	R227ea
LDPE	4.776667	0.050229	15.53723
PBT	0.444122	48.26917	0.322734
POM	0.91406	0.443895	8.34374
VINYL	0.339109	4.530149	0.286834

**Table 2:** Measured current as a function of flow rate for R152a propellant across different valve stem materials. The measured currents show the influence of valve material on triboelectric charging behaviour.

R152a							
POM		PBT		LDPE		VINYL	
Flow Rate (g/min)	Current (amp)	Flow Rate (g/min)	Current (amp)	Flow Rate (g/min)	Current (amp)	Flow Rate (g/min)	Current (amp)
24	123 pA	61	3.2 nA	53	1.3 nA	45	1.4 nA
52	681 pA	36	1.6 nA	74	4.1 nA	98	3.1 nA
85	1.1 nA	43	2.3 nA	127	7.6 nA	117	5.6 nA
153	1.7 nA	102	16.2 nA	27	233 pA	31	873 pA

**Table 3:** Physicochemical properties of the propellants used in this study, including molecular structure, relative permittivity, density, and viscosity.

Propellant	Common Name	Molecular Formula	Relative Permittivity	Density (kg/m^3^)	Viscosity (Pa·s)	Propellant Resistivity (Ω·m)
R152a	1,1-Difluoroethane	C_2_H_4_F_2_	1.004	899	0.00031	2.37 × 10^9^
R134a	1,1,1,2-Tetrafluoroethane	C_2_H_2_F_4_	1.15–1.22	1206.7	0.00050	3.02 × 10^10^
R227ea	1,1,1,2,3,3,3-Heptafluoropropane	C_3_HF_7_	1.25–1.29	1590.6	0.00076	1.31 × 10^10^

## Data Availability

The original contributions presented in this study are included in the article. Further inquiries can be directed to the corresponding authors.
